# A Case Report of Occult Breast Cancer Detected by Diagnostic Laparoscopy for Suspected Ovarian Cancer

**DOI:** 10.1155/2024/8851045

**Published:** 2024-04-26

**Authors:** Arisa Egami, Yosuke Tarumi, Ayaka Okamura, Kohei Aoyama, Hisashi Kataoka, Tetsuya Kokabu, Kaori Yoriki, Fumitake Ito, Taisuke Mori

**Affiliations:** Department of Obstetrics and Gynecology, Kyoto Prefectural University of Medicine, Graduate School of Medical Science, Kyoto, Japan

## Abstract

Diagnostic laparoscopy is useful in the management of gynecological cancers; however, it can occasionally result in the detection of other malignancies. Occult breast cancer (OBC) is metastatic breast cancer without a recognized primary breast lesion. We report a rare case of OBC that was detected laparoscopically. A 64-year-old female presented to our hospital with back pain. Magnetic resonance imaging (MRI) revealed a 50 mm multicystic tumor with an internal nodule in the right ovary. Positron emission tomography/computed tomography showed abnormal accumulation in multiple lymph nodes, moderate accumulation in the ovarian tumor nodule, and no accumulation in the breasts. Ovarian cancer was suspected, and a diagnostic laparoscopy was performed. Laparoscopically, a cystic tumor in the right ovary and 10 mm nodule in the right round ligament were observed and partially resected. Immunohistopathologically, the nodules of the round ligament exhibited features consistent with those of breast cancer, but the ovarian tumor was a seromucinous borderline tumor. MRI revealed no breast lesions. Therefore, the malignancy was diagnosed as an OBC.

## 1. Introduction

Diagnostic laparoscopy is performed for diagnosis, tissue sampling, and determination of resectability for malignancies such as ovarian, fallopian tube, peritoneal, and unknown primary cancers. Even when the preoperative diagnosis is uncertain, it may be possible to identify the primary origin through observation and tissue sampling using laparoscopy. Gastrointestinal and gallbladder cancers [1], malignant mesotheliomas [2], and malignant lymphomas [3] can be diagnosed using laparoscopy. Therefore, gynecologists often perform laparoscopy to diagnose malignancies of unknown origin.

Occult breast cancer (OBC) is defined as the metastasis of breast cancer to regional lymph nodes or distant organs without a primary breast lesion. OBC reportedly accounts for 0.3–1.0% of all breast cancers [4]. Currently, the diagnosis of OBC is based on a negative breast lesion on clinical and radiological examinations and pathological and immunohistochemical findings consistent with breast cancer [5]. OBC is primarily observed in patients with axillary lymphadenopathies. Intra-abdominal lesions are rarely detected, with no reports of OBC diagnosed laparoscopically.

Here, we report a rare case in which diagnostic laparoscopy was performed for suspected ovarian cancer, leading to a diagnosis of OBC and seromucinous borderline tumor of the ovary.

## 2. Case Presentation

**Figure 1:**
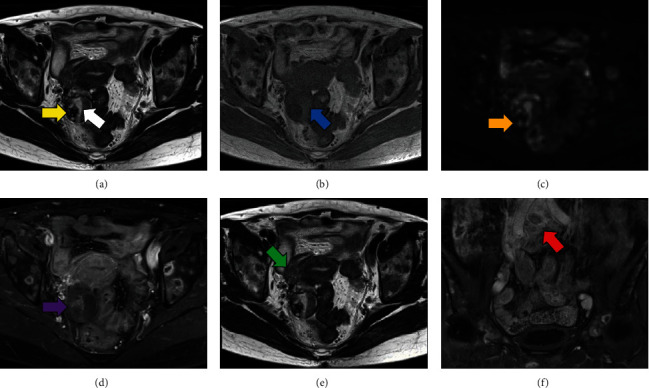
MRI findings. (a) MRI shows a 50 mm multicystic tumor with an internal nodule 15 mm in diameter in the right ovary. The fluid on the dorsal side of the tumor indicates high intensity (white arrow), and the nodule with multiple cysts shows low intensity (yellow arrow) in T2-weighted image. (b) The fluid on the dorsal side of the tumor indicates high intensity in T1-weighted image (blue arrow). (c) DWI shows partially reduced diffusion of the nodule (orange arrow). (d) Contrast-enhanced imaging shows heterogeneously enhanced contrast of the tumor (purple arrow). (e) The nodule in the right round ligament in T2-weighted image (green arrow). (f) Swollen lymph nodes of the pelvis in contrast-enhanced T1-weighted imaging (coronal plane, red arrow). MRI: magnetic resonance imaging; DWI: diffusion-weighted image.

**Figure 2:**
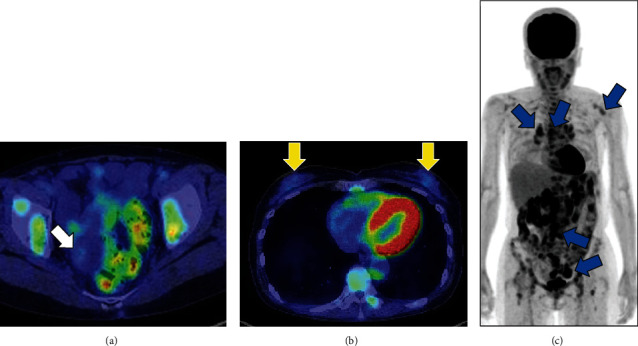
PET-CT findings. (a) PET/CT shows moderate FDG accumulation in the nodule of the right ovarian tumor (white arrow). (b) No significant FDG accumulation can be observed in mammary glands (yellow arrows). (c) Abnormal FDG accumulation can be observed in the lymph nodes of the supraclavicular, mediastinum, hilar, para-aorta, and pelvic regions (blue arrows). PET/CT: positron emission tomography/computed tomography; FDG: F-18 fluorodeoxyglucose.

**Figure 3:**
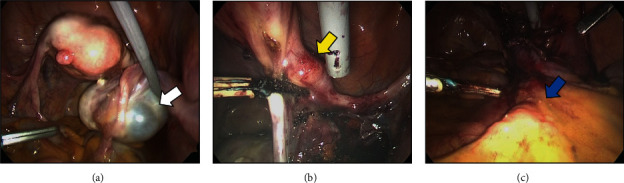
Operative findings. (a) 50 mm cystic tumor in the right ovary (white arrow). (b) 10 mm nodules in the right round ligament (yellow arrow). (c) Enlarged lymph nodes can be observed at the cranial side of the right common iliac artery bifurcation (blue arrow).

**Figure 4:**
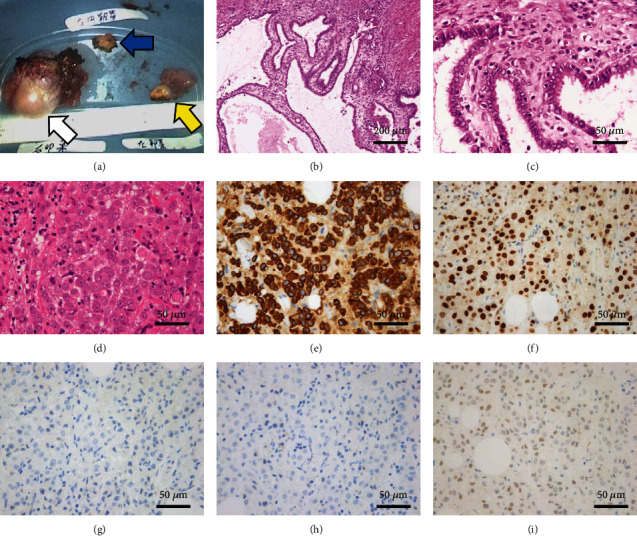
Resected specimens and histopathological findings. (a) Right adnexa (white arrow), left adnexa (yellow arrow), and the nodule of the right round ligament (blue arrow) were resected laparoscopically. (b, c) Histopathological examination shows that the ovarian tumor is a seromucinous borderline tumor. Papillary structure with serous, endocervical-type mucinous, and ciliated cells with mild nuclear atypia was found in the nodule of the cyst. (d) Adenocarcinoma tissues can be observed in the right round ligament and mesenteric nodules. Adenocarcinoma tissues are (e) GATA3 positive, (f) CK7 positive, (g) CK20 negative, (h) ER negative, and (i) PgR weakly positive. GATA3: GATA-binding protein; CK: cytokeratin; ER: estrogen receptor; PgR: progesterone receptor.

**Figure 5:**
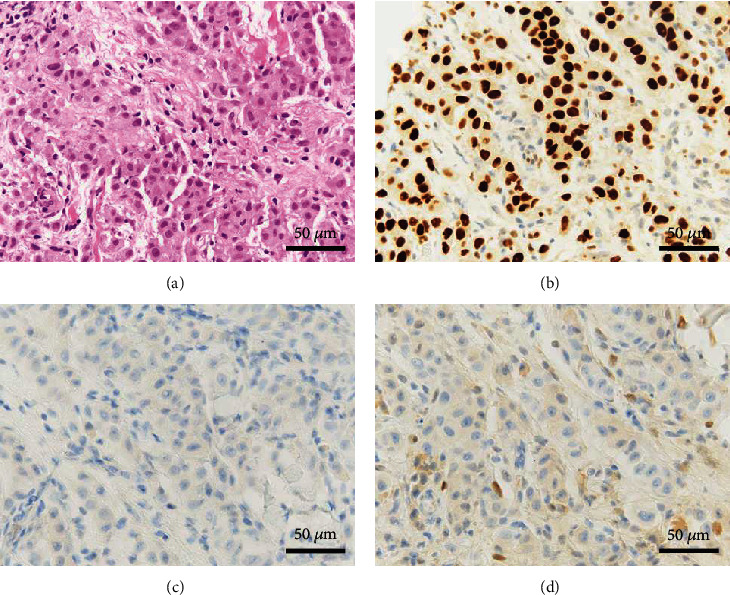
Histopathological findings of lymph node biopsy. (a) Adenocarcinoma tissues consistent with the tissues derived from the right round ligament and mesenteric nodules. Adenocarcinoma tissues were (b) GATA3 positive, (c) PAX-8 negative, and (d) ER-positive. GATA3: GATA-binding protein; ER: estrogen receptor.

A 64-year-old, gravida 2 para 2 female presented with back pain. She had no other significant past medical history. Transvaginal ultrasonography and magnetic resonance imaging (MRI) revealed a 50 mm multicystic tumor in the right ovary with a 15 mm internal nodule. The fluid on the dorsal side of the tumor showed high intensity on T1-weighted image and low intensity on T2-weighted image, suggesting that the fluid was bloody content (Figures 1(a) and 1(b)). The nodule with multiple cysts showed low intensity on T2-weighted image, reduced diffusion, and heterogeneously enhanced contrast (Figures 1(a), 1(c), and 1(d)). Additionally, MRI revealed the nodule in the right round ligament and swollen lymph nodes of the pelvis (Figures 1(e) and 1(f)). Positron emission tomography (PET)/computed tomography (CT) showed moderate F-18 fluorodeoxyglucose (FDG) accumulation in the nodule of the right ovarian tumor (SUV max: 1.79) (Figure 2(a)); abnormal accumulation in multiple lymph nodes, including the mediastinum, hilar, para-aorta, pelvic, and supraclavicular regions (SUV max: 6.96); and multiple bone metastasis including vertebrae (cervical to lumbar), ilium, and pubis. No notable accumulation was observed in either mammary gland (Figures 2(b) and 2(c)). Tumor markers were elevated: cancer antigen (CA) 125 47.2 U/mL, human epididymis protein 4 (HE4) 208.0 pmol/L, carcinoembryonic antigen (CEA) 29.8 ng/mL, and CA19-9 1021 U/mL. Gastroscopy and colonoscopy revealed no malignancy. We suspected ovarian cancer, stage IVB (FIGO2014), and performed diagnostic laparoscopy. Surgical findings revealed a 50 mm cystic tumor in the right ovary (Figure 3(a)), 10 mm nodules in the right round ligament (Figure 3(b)) and mesentery of the small intestine, and swollen lymph nodes of the para-aorta and right common iliac artery (Figure 3(c)). No disseminated lesions were observed in the peritoneal cavity. Bilateral salpingo-oophorectomy and right round ligament resection were performed (Figure 4(a)). Considering the possibility of malignant lymphoma, we also performed a CT-guided retroperitoneal lymph node biopsy. Histopathological examination revealed a seromucinous borderline tumor in the right ovary (Figures 4(b) and 4(c)). Conversely, adenocarcinoma tissue was detected in the right round ligament (Figure 4(d)). Immunohistochemical staining of the adenocarcinoma showed GATA3(+), PAX8(-), CK7(+)/CK20(-), TTF-1(-), and calretinin(-) (Figures 4(e)–4(g)). The estrogen receptor (ER) showed negative with total score (TS) 2, comprising proportion score (PS) 1 and intensity score (IS) 1 (Figure 4(h)). The progesterone receptor (PgR) showed weakly positive with TS of 5, comprising PS 4 and IS 1 (Figure 4(i)). The HER2 expression was scored 2+. Additionally, dual color in situ hybridization found negative for HER2 gene amplification by HER2/CEP17 ratio (1.05 (<2.0)) and average HER2 copy number (2.1 (<4.0)). These findings suggested that the malignancy was triple negative breast cancer. Retroperitoneal lymph node biopsy specimens showed adenocarcinoma with an immunohistochemical pattern similar to that of breast cancer (Figures 5(a)–5(d)). Although pathological and immunohistochemical findings were strongly consistent with the origin of this malignancy as breast cancer, postoperative breast MRI exhibited no malignant findings in the mammary glands. Furthermore, PET/CT confirmed metastasis in the supraclavicular fossa lymph nodes, which are the regional lymph nodes in breast cancer. Therefore, we diagnosed the malignancy as OBC, cT0pN3M1, triple negative status (negative ER, weakly positive PR, and negative HER2 status). Postoperatively, TS-1 followed paclitaxel (90 mg/m^2^), and bevacizumab (10 mg/kg) treatment was performed. The seromucinous borderline tumor of the right ovary was completely resected, warranting no additional treatment. The patient is alive 17 months after the operation.

## 3. Discussion

Diagnostic laparoscopy is useful for the diagnosis and tissue sampling of gynecological cancers and other malignancies. The National Comprehensive Cancer Network recommends laparoscopy for clinical staging and tissue biopsy in advanced ovarian cancer [6, 7], and its use has increased in clinical practice. Compared with exploratory laparotomy, laparoscopy shortens the operation time and reduces blood loss and the risk of perioperative complications [8]. However, several case reports of cancers whose primary lesion was unknown have been diagnosed using laparoscopy, including gastrointestinal and gallbladder cancers, malignant mesothelioma, and malignant lymphoma [1–3]. Gynecologists should cautiously evaluate the pathological findings of cancers of unknown primary origin for diagnosis. In the current case, the ovarian tumor, nodules in the right round ligament and mesentery of the small intestine, and retroperitoneal lymphadenopathy were confirmed on clinical examination. Initially, we suspected advanced ovarian cancer and performed a diagnostic laparoscopy. However, pathological findings revealed adenocarcinoma tissues distinct from the ovarian tumor, leading to a diagnosis of OBC.

OBC is defined as a carcinoma of unknown primary origin, consistent with metastatic carcinoma originating from the breast but without clinical findings in the breast itself. Radiological examination of the breast or a mastectomy is necessary to diagnose OBC. Previous studies on OBC were based on heterogeneous cohorts from population databases that included patients who may or may not have undergone MRI or pathological examination after mastectomy. The American College of Radiology recommends the use of MRI for patients with OBC to confirm the absence of primary breast cancer rather than mammography or ultrasound because of its sensitivity. MRI has been reported to identify a primary in 72% of cases that were deemed occult. Previous reports revealed that among 13 cases who had negative MRI findings, 2 cases (15.4%) were detected for primary lesion by mastectomy [5]. Positron emission mammography (PEM) has been employed as a useful investigatory tool with FDG to detect smaller tumors, especially those less than 1 cm in size. Although PEM has high specificity, MRI affords greater sensitivity than PEM for detecting breast lesions [9]. Although MRI is a rational method for identifying the absence of clinical findings in the breast itself, there remains no definition yet as to whether MRI, mastectomy for histological search, or both are necessary to diagnose OBC. The primary origin of OBC remains unknown. Previous report revealed that breast surgery including radical mastectomy and breast-conserving surgery combined with radiotherapy was associated with low mortality rate rather than axillary lymph node dissection or sentinel lymph node biopsy only, suggesting the unrecognized primary breast cancer [10]. Another paper also reported the better prognosis of mastectomy rather than breast-conservative surgery for OBC [11]. In contrast, another paper has reported that occult breast cancer may originate from ectopic breast tissue in axillary lymph nodes [12]. Breast cancer tissue in OBC is often diagnosed primarily by biopsy of lymph nodes, such as the axillary lymph nodes [5]. Conversely, several cases of OBC have been diagnosed by examining distant metastatic lesions, such as those of the thyroid gland, skin, and bone [13–15]. In the current case, postoperative MRI and PET/CT confirmed the absence of breast lesions, and the adenocarcinoma tissues resected using diagnostic laparoscopy were strongly consistent with breast cancer. These findings fulfilled the diagnostic criteria for OBC, and mastectomy was not performed. Patients with OBC tend to have a high age at diagnosis, nodal stage N3, and ER(-)/PgR(-) and HER2(+) on immunohistopathological assessment [16]. Current reports of 33 cases of OBC have reported that the percentages of ER-positive, PgR-positive, and HER2-positive status were 51.5%, 54.5%, and 72.7%, respectively [17]. This case also revealed nodal stages N3, ER(-)/PgR(weakly +), and HER2 negative. Although a previous study has reported that the 10-year overall survival rate of OBC was similar to that of non-OBC, varying from 47.5 to 68.8%, the prognosis remains controversial [16, 18].

The pathological and immunohistochemical findings of metastatic lesions are important for the diagnosis of OBC. Morphologically, OBC frequently exhibits features resembling adenocarcinomas or undifferentiated carcinomas, characterized by infiltrating malignant epithelial cells with variable glandular, tubular, and solid architectures. A combination of CK7 and CK20 expression profiling is useful for identifying various primary tumor sites. For example, CK7(+)/CK20(-) profiling can suggest the origin of breast, lung, mesothelial, ovarian, and endometrial carcinomas. Organ-specific markers are also useful for this purpose. Examples include the breast (ER, PgR, mammaglobin, and GCDFP), ovary (PAX8, WT-1), thyroid (TTF-1, PAX8, and thyroglobulin), mesothelium (calretinin), and lungs (TTF-1, napsin A) [19]. However, OBC reportedly exhibits a tendency toward ER/PgR-negative expression [16]. GATA-3, an important transcription factor involved in cell proliferation and differentiation, has recently been recognized as a promising marker of breast carcinoma. Ni et al. have reported that GATA-3 shows higher sensitivity (82.5%) than mammaglobin (46.6%) and GCDFP15 (23.9%) for detecting metastatic breast cancer [20]. In the current case, the immunohistochemical profile of CK7(+)/CK20(-), ER(-)/PgR(weakly +), GATA3(+), PAX8(-), TTF-1(-), and calretinin(-) was concordant with findings in OBC.

Minimally invasive surgery, such as diagnostic laparoscopy, is generally beneficial because of its shorter hospitalization period, decreased rates of perioperative complications, wound infection, postoperative pain, and quicker recovery compared with laparotomy [21]. However, a case of port site metastasis of breast cancer after diagnostic laparoscopy has been reported [22]. It should take care to administer diagnostic laparoscopy.

OBC is a rare malignancy with no detectable primary lesions. When laparoscopically biopsied metastatic tumor had histologic features consistent with breast cancer, in the absence of a recognized breast lesion, the possibility of OBC should be considered.
